# Syntaxin 5 Overexpression and *β*-Amyloid 1–42 Accumulation in Endoplasmic Reticulum of Hippocampal Cells in Rat Brain Induced by Ozone Exposure

**DOI:** 10.1155/2016/2125643

**Published:** 2016-06-05

**Authors:** Luis Fernando Hernández-Zimbrón, Selva Rivas-Arancibia

**Affiliations:** Departamento de Fisiología, Facultad de Medicina, Universidad Nacional Autónoma de México, 04510 México, DF, Mexico

## Abstract

Oxidative stress is a risk factor for Alzheimer's disease and it is currently accepted that oxidative damage precedes the overproduction of A42 peptide. We have reported that ozone causes oxidative stress inducing neurodegeneration in the brain of rats. It is associated with A42 overproduction and intracellular accumulation in hippocampus. Organelles like mitochondria, intracellular membranes, and endoplasmic reticulum have been identified as sites of A42 production and accumulation affecting cellular metabolism. However whether ozone exposure induces overproduction and/or accumulation of A42 in endoplasmic reticulum has not been studied. We evaluated this effect in the endoplasmic reticulum of hippocampal cells of rats exposed chronically to low doses of ozone (0.25 ppm) at 7, 15, 30, 60, and 90 days. The effect of the presence of A42 in endoplasmic reticulum was analyzed evaluating the expression of the chaperone Syntaxin 5. Our results show an accumulation of A42 peptide in this organelle. It was observed by immunofluorescence and by WB in endoplasmic fractions from hippocampal cells of rats at 60 and 90 days of treatment. Significant overexpression of the chaperone Syntaxin 5 at 60 and 90 days of treatment was observed (^⁎^ *P* < 0.05). These results indicate that the exposure to environmental pollutants could be involved as a risk factor for neurodegenerative processes.

## 1. Introduction

Oxidative stress is produced as a consequence of pathological, dietary, and environmental factors. Many people living in large cities around the world are affected by environmental pollutants like ozone (O_3_) and it has been demonstrated that chronic exposure to ozone (0.025 ppm) similar to that reported in a day of high pollution in Mexico City causes a state of oxidative stress [1]. Oxidative stress is a major risk factor for Alzheimer's disease (AD). Besides, it has been shown that free radicals enhance the amyloid pathology of AD [2].

Several previous studies from our laboratory have shown that the chronic oxidative stress caused by O_3_ produces progressive neurodegeneration in rat hippocampi. In addition memory deterioration, motor activity deficits, lipid peroxidation, and mitochondrial dysfunction were also observed in the hippocampi [3–7].

More recently we have shown a direct relationship of *β*A42 overproduction and intracellular accumulation with oxidative stress [6, 8].

Many reports have determined that the extracellular and intracellular accumulation of *β*A42 is involved in the development and progression of AD [8]. However, the precise mechanism of *β*A42 neurotoxicity is not completely understood.

It has been demonstrated that mitochondria, ER, and Golgi apparatus are targets of *β*A42, while ER dysfunction in AD is well documented [2, 6, 8–12]. However, whether oxidative damage precedes and contributes directly to the ER accumulation of the *β*A42 peptides remains unclear.

Previous studies have shown that *β*A42 can bind to different proteins and extracellular and intracellular macromolecules that affect normal neuronal function and it has been proposed that ER dysfunction is a distinctive hallmark in AD. For example, the presence of *β*A42 in ER causes ER stress which in turn activates indicators of the unfolded protein response (UPR), a response activated by ER stress. This kind of alterations affects cytoskeleton integrity and leads to cell death by *β*A42 presence in neurons [13]. Besides, the accumulation of *β*A42 induces apoptosis by activating different pathways as the crosstalk between ER and mitochondria. On the other hand mitochondrial dysfunction triggers caspases 3, 8, and 12 activation in cybrid cells, indicating that the death receptor and the ER-specific apoptotic pathways are also activated upon exposure to *β*A42 [14–16].

It is well known that the mitochondrial pathway is one of the principal generators of oxidative stress. Nevertheless, the stress in the ER is involved or may be induced by oxidative stress in AD. For example, disturbances of calcium and ER homeostasis induce an oxidative stress state and potential *β*A42 aggregation mediated by iron [17].

Our aim in this work was to demonstrate that the chronic exposure of rats to an environmental pollutant like O_3_ (0.25 ppm) may cause an imbalance in the production of *β*A42. Such imbalance may lead to the progressive accumulation of this peptide in the ER, inducing changes in the normal metabolism of the cells.

## 2. Materials and Methods

### 2.1. Animal and Animal Care

72 male Wistar rats weighing 250–300 g were individually housed in acrylic boxes within a clean air box, and food was provided* ad libitum* (NutriCubo, Purina, USA). The control and treated rats were maintained in a temperature-controlled and humidity-controlled environmental bioterium. The animals were maintained and treated in accordance with the Norma Official Mexicana NOM-036-SSA 2-2002 and the Bioethics Committee of the Faculty of Medicine at the National Autonomous University of Mexico.

### 2.2. General Procedure

The rats were randomly separated into six experimental groups (*n* = 12 per group). Group 1 was exposed daily to a clear airstream free of O_3_ for 4 h, and groups 2, 3, 4, 5, and 6 were exposed to O_3_ for 7, 15, 30, 60, and 90 days, respectively. The experimental groups were exposed to 0.25 ppm of O_3_ for 4 h daily. One of these subgroups was used for immunohistochemical analyses, and the other group was used for cellular fractionation.

### 2.3. O_3_ Exposure

In order to expose them to chronic doses of ozone (0.025 ppm) the animals were placed inside a chamber and the procedure was carried out essentially as described [7].

Two hours after the final exposure to clean air or O_3_, the animals from each group were anesthetized with sodium pentobarbital (50 mg/kg i.p.; Sedalpharma, Edo. de México, Mexico) and then decapitated.

The hippocampi of six animals from each group were obtained for Western Blot (WB), and the other three animals were transcardially perfused with 4% paraformaldehyde (Sigma-Aldrich Chemie, Germany) in 0.1 M phosphate buffer (J.T. Baker, NJ; PB, Tecsiquim; pH 7.4) for the immunohistochemistry assays. The postfixation of the brains was made essentially as described previously [1]. Five-micrometer sagittal slices of the brain containing the hippocampus were obtained using a microtome (American Optical), mounted on slides, and stored.

### 2.4. Subcellular Fractionation

To isolate the endoplasmic reticulum fractions, the hippocampal cells were lysed with a Dounce homogenizer. Microsomes from these cells were isolated using the Endoplasmic Reticulum Isolation Kit (Sigma-Aldrich) according to the manufacturer's instructions. The ER fractions were used immediately for WB assays as described by Hernández-Zimbrón and Rivas-Arancibia [7].

### 2.5. Western Blot (WB)

The production levels of *β*A42 in the RE fractions were analyzed by gel electrophoresis and WB. The tissue was homogenized, and 50 *μ*g of protein from each sample was boiled and separated on a 4–12% SDS polyacrylamide gel (Invitrogen) for 45 min at 90 volts (V). The proteins were electrophoretically transferred onto PVDF membranes (Sigma-Aldrich, San Luis, MO, USA). The membranes were blocked with 4% fat-free milk in Tris buffer solution (TBS-T) with 0.01% Tween 20 (TBS-T) (Sigma-Aldrich, San Luis, MO, USA) overnight at 37°C. After being blocked, the membranes were incubated individually with the following antibodies: rabbit anti-*β*A42 (Abcam Inc., Boston, MA, USA) (1 : 2000). The anti-GRP78 antibody (ER marker) was used as loading control. To evaluate the effect of the presence of *β*A42 in the ER, the expression levels of Syntaxin 5 (Syx5) were evaluated and the antibody goat anti-Syntaxin 5 (Santa Cruz Biotechnology, CA, USA) was used (1 : 200).

The membranes were incubated overnight with gentle shaking at 4°C (Brinkmann OrbMix 110, Brinkmann, Germany). The membranes were rinsed with TBS-T and subsequently incubated for 2 h at room temperature (RT) in TBS-T containing goat anti-rabbit IgG conjugated to the horseradish peroxidase secondary antibody and diluted to 1 : 1000 (Santa Cruz Biotechnology, CA, USA) for 1 h. The immunoreactive bands were detected by chemiluminescence (ECL; General Electric, Santa Clara, CA, USA). The intensity of each band was quantified using an imaging densitometer (model GS-700). The intensities were analyzed using the open access ImageJ software (NIH) as we previously reported.

### 2.6. Double Immunofluorescence for *β*A42

Rabbit monoclonal anti-*β*A42 antibody (obtained from Abcam, MA, USA), Goat polyclonal anti-GRP78 (Santa Cruz Biotechnology, CA, USA), and goat polyclonal anti-Syx5 (Santa Cruz Biotechnology, CA, USA) were used for double IF to detect the *β*A42 peptide in the ER of hippocampal cells of the rats.

Sagittal sections of each brain containing the hippocampus were paraffin-embedded, treated with a paraffin-removal and heat-retrieval solution (Biocare Medical), and placed in a Decloaking Chamber (Biocare Medical) for 5 min. Then, the sections were rinsed with distilled water and treated with a blocking reagent (PBS-Bovine Serum Albumin (0.04%)) for 1 hour, washed with 0.1 M phosphate saline buffer, and incubated individually for 12 h at 4°C with anti-*β*A42 (dilution 1 : 200).

For the double immunofluorescence assays (IF), all primary antibodies were incubated overnight at 4°C and then rinsed three times with PBS/Triton (0.03%) and incubated for 2 hours with secondary antibodies. Rabbit monoclonal anti-*β*A42 and goat polyclonal anti-GRP78 (dilution 1 : 200) were used and visualized with Alexa Fluor 488 goat anti-rabbit IgG (H+L) and Alexa Fluor 594 donkey anti-goat IgG (H+L), respectively. Rabbit monoclonal anti-*β*A42 and goat polyclonal anti-Syx5 (dilution 1 : 200) were used and visualized with Alexa Fluor 594 goat anti-rabbit IgG (H+L) and Alexa Fluor 488 mouse anti-goat IgG (H+L). All the secondary antibodies were from Molecular Probes, OR, USA. Finally, the slides were rinsed and mounted onto glass slides in Vectashield medium (Vector Laboratories, Burlingame, CA, USA) containing 4′,6-diamino-2-phenylindole (DAPI). Representative brain sections from each group were processed in parallel afterwards and these sections were observed through a Leica DM-LS epifluorescence microscope at 40x and 100x (Leica Microsystems, Wetzlar, GmbH, Germany). The fluorochromes were visualized with their specific filters and analyzed in three channels.

### 2.7. Statistical Analysis

All of the data are expressed as mean ± SEM. ANOVA analyses followed by Fisher's LSD post hoc, Bonferroni's, and Tukey's tests were used for multiple comparisons. Prism Graph Pad Software was used (Systat Software, Inc., Point Richmond, CA, USA) and differences were considered significant at *P* < 0.05.

## 3. Results

### 3.1. *β*A42 Accumulation in RE Fraction under Oxidative Stress Conditions

**Figure 1:**
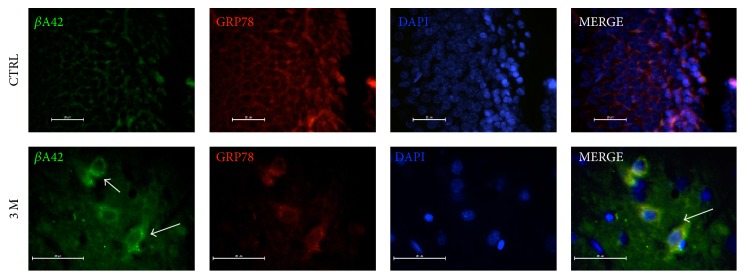
Triple IF to detect beta-amyloid 1–42 in the ER. Five micrometer-thick brain tissue sections form the hippocampi of the control group (CTRL) and the rats exposed to O_3_ (0.025 ppm) for 3 months (3 M) are shown. Goat anti-GRP78 was used as ER marker. Rabbit anti-*β*A42 is shown in green and GRP78 in red. DAPI staining and merge between the red, green, and blue channels are shown (MERGE). The arrows indicate the intracellular *β*A42 deposition and colocalization in ER. There was no *β*A42 detection at 7, 15, 30, and 60 days of treatment (data not shown).

We have reported that oxidative stress state caused by O_3_ exposure induces intracellular *β*A42 accumulation as well as mitochondrial accumulation in our neurodegeneration model [6, 7]. To prove the accumulation of this peptide in ER of hippocampal cells of rats treated with low doses of O_3_, we performed double IF assays in brain sections derived from both control and experimental groups. The double IF assays showed qualitative increases in the intrareticular accumulation of *β*A42 in the hippocampal dentate gyrus cells only at 90 days (3 M) of treatment (Figure 1).

### 3.2. Accumulation of *β*A42 in ER Isolated Fractions Caused by O_3_ Exposure

**Figure 2:**
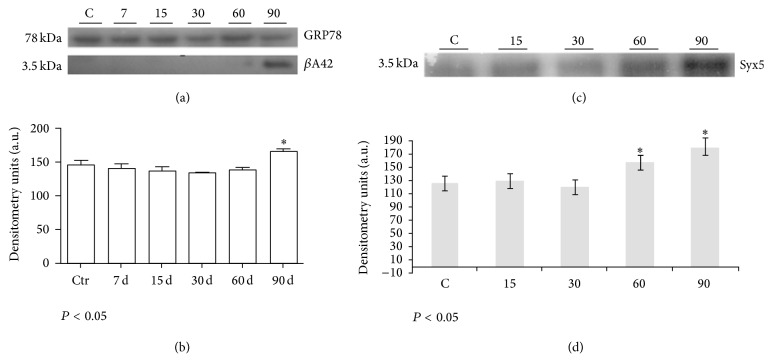
Immunodetection of *β*A42 and Syx5 in the ER protein extracts of rat hippocampi following different O_3_ treatments. (a) WB for *β*A42 in the ER fractions. The ER marker GRP78 was used as loading control and ER isolation control. (b) Densitometry analyses of the WB assays for *β*A42. *β*A42 production increased by the end of the ozone treatment. The differences became statistically significant at 90 days of O_3_ exposure for *β*A42; ^*∗*^ *P* < 0.05. (c) WB for Syntaxin 5 from the ER fraction (control, 15, 30, 60, and 90 days). Rabbit anti-Syntaxin 5 was used for immunodetection and was visualized by chemiluminescence. (d) Densitometry analyses of the WB assays. There were significant changes in Syntaxin 5 expression at 60 and 90 days of treatment. The differences between the control and experimental groups were statistically significant; ^*∗*^ *P* < 0.05.

Next, in order to demonstrate the accumulation of *β*A42 in the ER fractions, the level of *β*A42 was quantified by WB and densitometry analyses from the rat hippocampal cells isolated on days 0 (control), 7, 15, 30, 60, and 90 of exposure to O_3_. The most representative values of *β*A42 were obtained. As we can see in Figure 2(a), ~3.5 kDa band for the *β*A42 monomer was detected only at 90 days of ozone treatment, demonstrating *β*A42 accumulation in the isolated ER fractions when compared with the control group. The densitometry analysis showed a significant increase of *β*A42 accumulation in ER fractions (Figure 2(b)).

### 3.3. Oxidative Stress Causes Overexpression of Syx5 in ER

**Figure 3:**
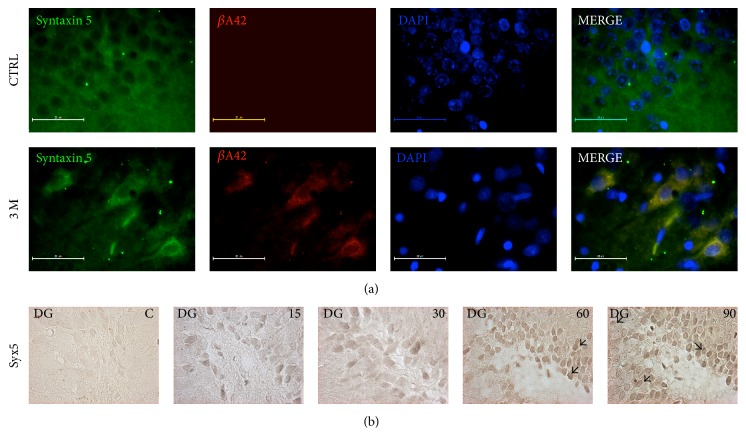
Immunodetection of Syx5 and *β*A42 in the ER. Five micrometer-thick brain tissue sections form hippocampi of the control group (CTRL) and the rats exposed to O_3_ (0.25 ppm) for 3 months (3 M) are shown. Rabbit anti-*β*A42 is shown in red and Syntaxin 5 in green. DAPI staining (DAPI) and merge between red, green, and blue channels are shown (MERGE). The arrows indicate the overexpression of Syx5 and the intracellular *β*A42 deposition and colocalization in the ER (a). There was no *β*A42 detection at 7, 15, 30, and 60 days of treatment (data not shown). (b) Immunohistochemistry for Syx5 in brain slides of control (C), 15, 30, 60, and 90 days of treatment showing the overexpression of Syx5 under O_3_ treatment.

To demonstrate the possible effect of oxidative stress and beta-amyloid accumulation in ER metabolism, we have performed WB and IF assays to detect changes in the expression levels of Syx5 protein. Syx5 protein has been related with communication processes between mitochondria and ER and described as a chaperone as well. Our WB results showed that ozone exposure alters the expression of Syx5 at 60 and 90 days (Figures 2(c) and 2(d)). Besides, the overexpression of Syx5 was observed by IF (Figure 3(a)) at 90 days and by immunohistochemistry principally at 60 and 90 days of treatment (Figure 3(b)).

## 4. Discussion

In this study we have demonstrated that O_3_ exposure (0.25 ppm) induced a significant increase of Syntaxin 5 and accumulation of *β*A42 peptide in the ER in cells of the dentate gyrus. Changes in the expression patterns of the chaperone Syntaxin 5 precede the accumulation of *β*A42 after the treatment with this environmental pollutant.

AD is a neurodegenerative disease with a complex and progressive pathological phenotype [18]. The *β*A42 peptide, a hallmark of AD, is produced through the sequential cleavage of APP by *β*- and *γ*-secretase [19]. Several reports have demonstrated that the origin and progression of AD are highly correlated with oxidative stress status. Oxidative damage, mitochondrial dysfunction, and age dependent increases of reactive oxygen species (ROS) have been identified as key factors in the development of sporadic AD [20–22]. Besides, oxidative stress may contribute to neuronal degeneration in AD by increasing iron and lipid peroxidation, protein oxidation, and some markers of oxidative stress that are present in the senile plaques in AD [23–25].

Previous results from our laboratory and other groups have demonstrated that the chronic administration of O_3_ (0.25 ppm) induces an oxidative stress status, increased lipid peroxidation levels in different brain structures, and morphological and structural changes in neurons and elevates hippocampal superoxide accumulation [26–29].

More recently, we demonstrated that rats exposed to O_3_ overproduce *β*A42, inducing intramitochondrial *β*A42 accumulation [7]. Moreover, ER oxidative damage has also been well documented in AD. It is well known that the mitochondrial pathway is the main generator of ROS. ER stress is caused by ROS in AD, while the disturbances of calcium and ER homeostasis induce an oxidative stress state which increases Syntaxin 5 and lead to potential *β*A42 aggregation. However, the precise mechanism that links ER oxidative damage with abnormal *β*A42 overproduction and accumulation has not been elucidated [30].

Based on our previous results showing mitochondrial accumulation of *β*A42 we performed assays to demonstrate the effect of ozone exposure in ER. We found an increase of Syntaxin 5 before the accumulation of *β*A42 in the ER of hippocampal cells. Both effects could be related to ER metabolism disturbance in our progressive neurodegeneration model.

First, we suggest that the overproduction or the increase in *β*A42 accumulation in hippocampal cells could be related with the increase of ROS production; second, it could be related with the accumulation and processing of APP in these cells. As we have demonstrated before, there is an increase of the amyloidogenic pathway activity in mitochondria [7]. This overactivity and overproduction of beta-amyloid peptide could be associated with the accumulation in ER membranes, mitochondrial membranes, or Mitochondrial Associated Membranes (MAM) as has been suggested by Pereira, 2013 [31–36].

As we have mentioned before, ER is a system formed by continuous membranes. It comprises different architectural shapes of the nuclear envelope, sheet-like structures, containing polyribosomes and smooth tubules present throughout the cell. This organelle is involved in the synthesis, folding, structural maturation, control, and trafficking of integral membrane and secreted proteins in cells. According to its membranous structure, there is evidence demonstrating that MAM are physiological interactions between the ER and mitochondria and have different functions such as lipid transport and synthesis, ER calcium regulation, mitochondrial calcium release, mitochondrial movement and morphology, and protein trafficking.

It has been recently described that PS2 is a protein present in MAM and that the complete *γ*-secretase complex is present in both ER and MAM [32, 33, 37–40].

In accordance with our results that show the accumulation of *β*A42 in ER, we could suggest that the *β*A42 overproduced and PS1 overexpressed in mitochondria may be transported to ER through MAM. The overcleavage of APP by PS1 and PS2 in MAM has been reported in AD and under oxidative stress conditions, as it has been deeply reviewed by [37, 40]. It should be pointed out that the connectivity is increased between ER and mitochondria in AD, and it might be happening in the model present in this work [7, 32, 33]. Another feasible mechanism to explain the *β*A42 presence in ER is the oxidative stress caused by O_3_ exposure* per se*.

The increase in the production and accumulation of beta-amyloid peptide in hippocampal cells may be correlated with the significant increase in PS1 expression and a reduced expression of ADAM9 under oxidative stress conditions. This indicates that low doses of O_3_ elicited the overactivation of the amyloidogenic pathway [7].

Then, to demonstrate the effect of the accumulation of *β*A42 in ER, we evaluate Syntaxin 5, a chaperone directly related with the communication between ER and mitochondria. For example, Syntaxin 5 and Syntaxin 17 facilitate the transport of cholesterol to mitochondria [33, 34].

Syntaxins are a family of vesicular transport receptors involved in membrane traffic through both the constitutive and regulated secretory pathways [41]. One of Syntaxin 5 functions is to shape the ER and is expressed ubiquitously in many cell types and has been located in the ER and Golgi compartment of the early secretory pathways [42]. Besides, Syntaxin 5 is thought to regulate the potential targeting and fusion carrier vesicles at multiple membrane fusion interfaces by affecting the selective combination of the SNARE complex with other SNARE-related proteins (6–9). Some reports have demonstrated that Syntaxin 5 specifically interacts with PS holoproteins, but not with the N-terminal or C-terminal fragments of PS, and the overexpression was shown to upregulate *β*APP accumulation in the ER through the Golgi compartments, attenuate accumulation of the C-terminal fragment of *β*APP, and reduce A*β* secretion [16].

Our results show that O_3_ exposure induces the overexpression of the chaperone Syntaxin 5 at 60 and 90 days of treatment observed by WB (Figures 2(c) and 2(d)) and by IF and IMHQ (Figure 3).

**Figure 4:**
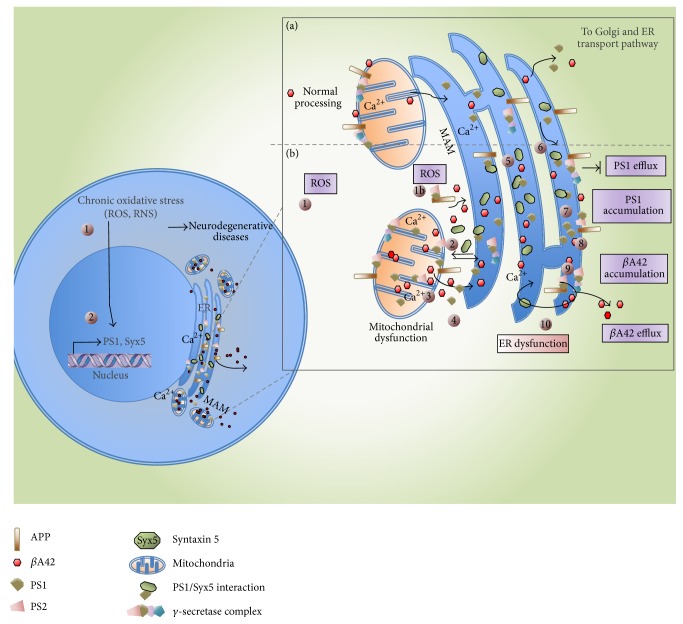
Schematic representation of the suggested mechanisms related with *β*A42 accumulation in ER. (a) Normal metabolism of mitochondria and ER under no oxidant stress conditions. Normal processing of *β*A42. (b) Metabolism alterations under oxidant stress conditions. (1) O_3_ induces an oxidative stress state causing PS1 and Syx5 overexpression. (1b) ROS induces by itself the overcleavage of APP by PS1 and PS2. (2) PS1 and Syx5 accumulate in mitochondria, MAM, and ER. (3) *β*A42 accumulation in mitochondria, MAM, and ER. (4) Transport of PS1, Syx5, and *β*A42 through MAM. (5) Syx5 excess in ER. (6) Interaction Syx5/PS1 blocks the PS1 normal efflux. (7) PS1 accumulation. (8) PS1 transport to *γ*-secretase complex. (9) Accumulation of *β*A42 in ER. (10) ER dysfunction.

Besides, it has been described that Syntaxin 5 interacts with PSs in ER and the Golgi compartment and its overexpression causes the accumulation of the PS and *β*APP as well as the reduction in the level of A*β* secretion [40, 42]. Then we could suggest that the accumulation of A*β* in ER could be in part a consequence of the overexpression of Syntaxin 5 (Figure 4).

As we mentioned before, PSs are located in the ER and although they lack proteolytic processing they are active molecules in AD with *γ*-secretase activity.

The increase of the expression of PS1 in the late compartment of the secretory pathways may lead to higher levels of *β*A40 and *β*A42 production [43]. We have reported that there is an overexpression of PS1 in the mitochondria of hippocampal cells at 60 and 90 days of O_3_ exposure [7]. We suggest that PS1 overexpressed may be transported to ER through MAM or a different mechanism. Then, PS1 and PS2 in the ER could bind to Syntaxin 5, inhibiting the degradation or transport through the ER associated pathway. In addition, it has been demonstrated that Syntaxin 5 in cooperation with PS alters the production of *β*A peptides. It affects the processing and/or trafficking of the fragment called *β*APP, which arrives at the site where *γ*-secretase appears.

Additionally, it has been demonstrated that Syntaxin 5 overexpression reduced the concentration of A*β*40 peptide secreted, a fact that supports our results and suggestions [44, 45].

Through the methodological approach that we have used in this work we cannot confirm that there are defects in the ER-Golgi transport; only we suggest this point based on the bibliographic review. For example, some authors suggest that it may be possible that perturbation of Syx5 isoform expression in neuronal cells cause some changes in A*β* production in the central nervous system. Suga et al. have demonstrated that Syx5 binds to the PS holoprotein in the ER and Golgi compartments of neuronal cells and they modulate the metabolism and trafficking of *β*APP. Because PS and *β*APP are sorted and processed along the secretory and endocytotic pathways, alterations in the transport machinery could affect the trafficking of these proteins, affecting the generation and secretion of A*β*. Thus, malfunction of this chaperone may cause the accumulation of excess A*β* peptides found in late-onset AD.

Finally, the ER stress and oxidative stress cause changes in the pattern of APP processing in affected neurons, increasing the amount of *β*A42 peptide. ROS also play a role in cell signaling through a mechanism known as redox signaling. Any alteration in the cellular antioxidant defense system and the increase in ROS lead to redox signaling alterations, which are involved in neurodegenerative processes and directly in the overproduction of *β*A42 [5–7, 46].

A*β* and oxidative stress together are able to trigger an ER stress response, leading to synaptic and neuronal loss, increasing the levels of markers of the ER stress response (UPR), and activating caspases pathway and apoptosis in cortical neurons [13, 15, 16, 36, 47]. Besides, the presence of A*β* in the ER induces the expression of some chaperones like GRP78 and GRP94, involved in the caspase-12 activation and cell death [48].

## 5. Conclusion

Cell injury due to ER stress has emerged as a key contributor to the pathophysiology of a wide range of neurodegenerative human diseases like Alzheimer's disease and pathological aging. Additional studies are needed to further investigate the impact of *β*A42 accumulation in the ER. Nevertheless, the present results help to show that the current levels of environmental pollutants in highly polluted cities might induce and participate in the development of neurodegenerative processes by oxidative stress.
